# Supplementary material for: Oscillation of p38 activity controls efficient pro-inflammatory gene expression
Source: Nat Commun. 2015 Sep 24;6:8350. doi: 10.1038/ncomms9350 (PMC4598561; doi:10.1038/ncomms9350)
Supplement: Supplementary Information — Supplementary Figures 1-11 [file ncomms9350-s1.pdf]

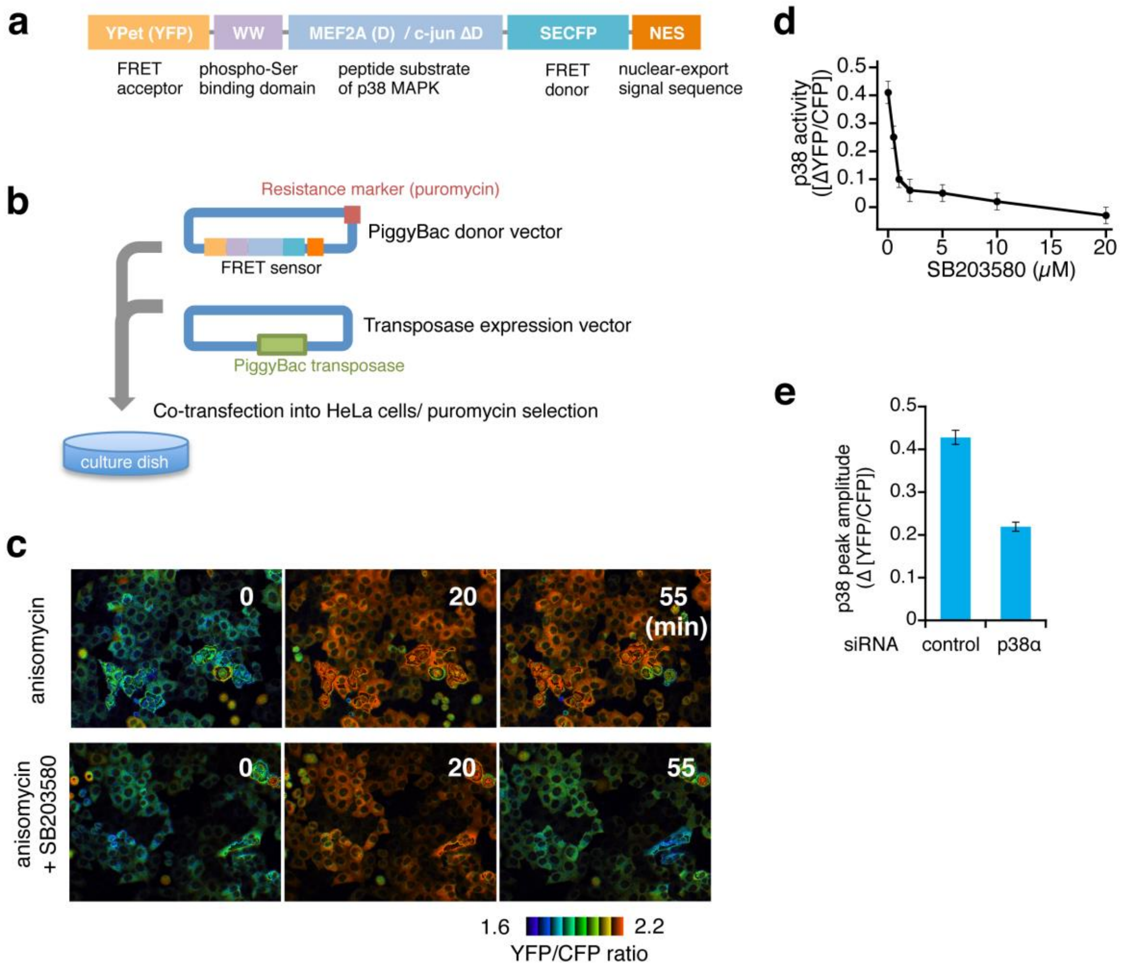

Supplementary Figure 1. Procedures for p38 activity imaging in living cells.

(**a**) Schematic model of the p38 activity reporter. The reporter consists of: (i) the YPet domain (an enhanced YFP); (ii) the Pin1 WW domain; (iii) a synthetic peptide that contains the D-domain (the p38-docking site) of MEF2A (amino acid residues 266-291: MNSRKPDLRVVIPPSSKGMMPPLNTQ) and the Ser-Pro site of c-jun (amino acid residues 54-73: RAKNSDLLTSPDVGLLKLAS); (iv) the SECFP domain (an enhanced CFP); and (v) a nuclear export sequence (LQKKLEELELDE).

(**b**) HeLa cells that stably express the p38 reporter were established using the PiggyBac transposon gene transfer system.

(**c**) FRET signal (YFP/CFP ratio) of the p38 reporter cells stimulated with anisomycin (10 ng ml^{-1}). The p38 inhibitor SB203580 (25 μM) was added 20 min after anisomycin.

(**d**) Anisomycin-induced FRET signal of the p38 reporter cells was inhibited by various concentrations of SB203580. Error bars indicate SEM (n=25 cells per each data point).

(**e**) FRET responses in p38 reporter cells transfected with either control siRNA or p38α-targeting siRNA. At 48 h after transfection, reporter cells were stimulated continuously with IL-1β (10 ng ml^{-1}). Peak signal amplitude, which occurred between 15 to 90 min after addition of IL-1β, was quantified. Error bars indicate SEM (n=20 cells).

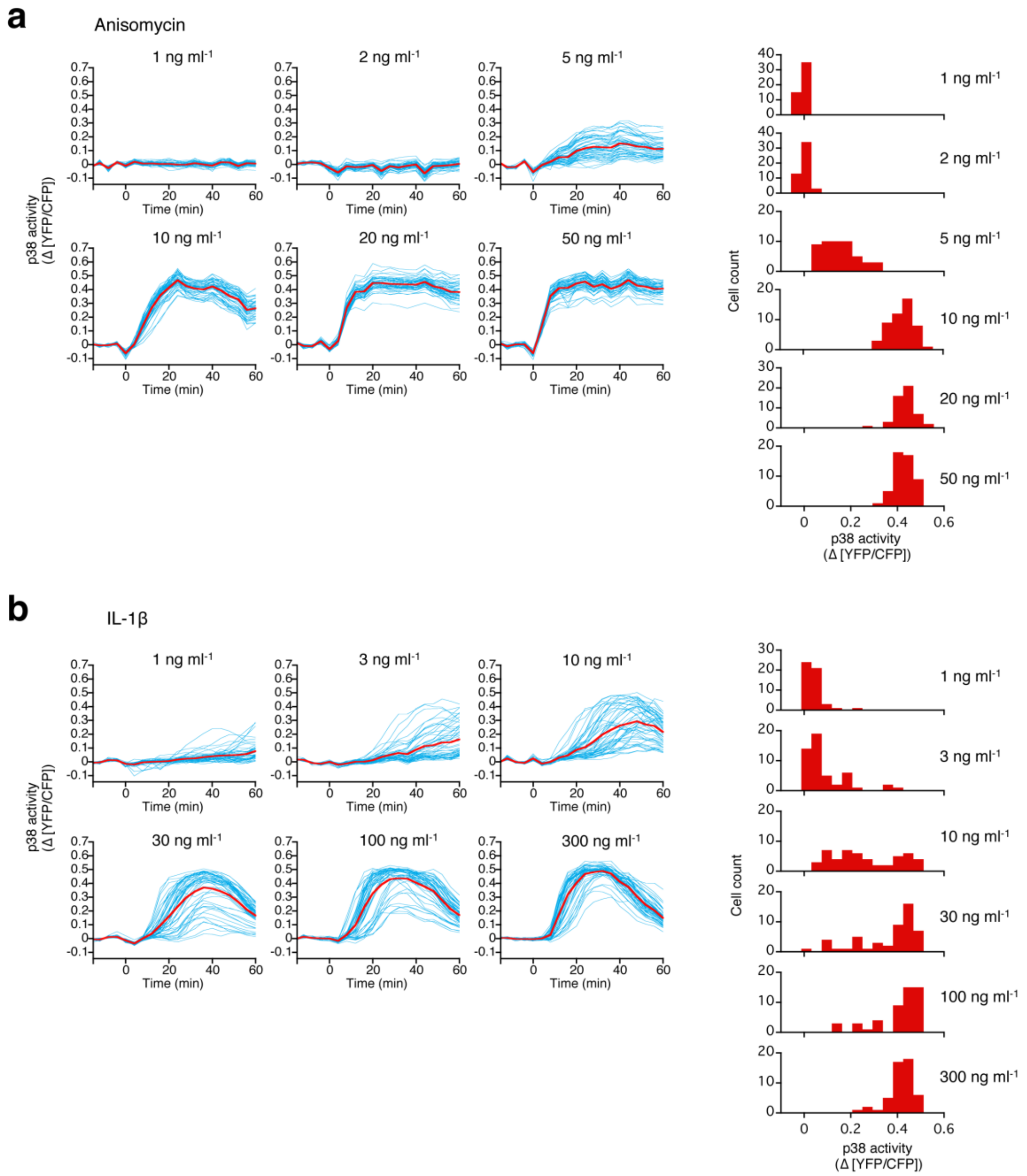

Supplementary Figure 2. Response of p38 to short-term application of different doses of activators. (**a and b**) Time-courses of p38 activation in individual p38 reporter cells. Cells were continuously stimulated with various doses of anisomycin (**a**) or IL-1β (**b**). Cyan lines, p38 activity responses of individual cells; red lines, the average values. Histograms at right indicate the distribution of p38 activity at 40 min (n=50 cells per group).

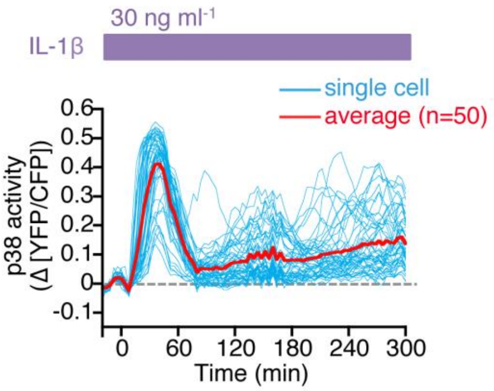

Supplementary Figure 3. p38 response to continuous IL-1β stimulation over five hours. p38 reporter cells were continuously stimulated with IL-1β (30 ng ml^{-1}). Responses of individual cells are shown by cyan curves, and the average trace is in red (n=50 cells).

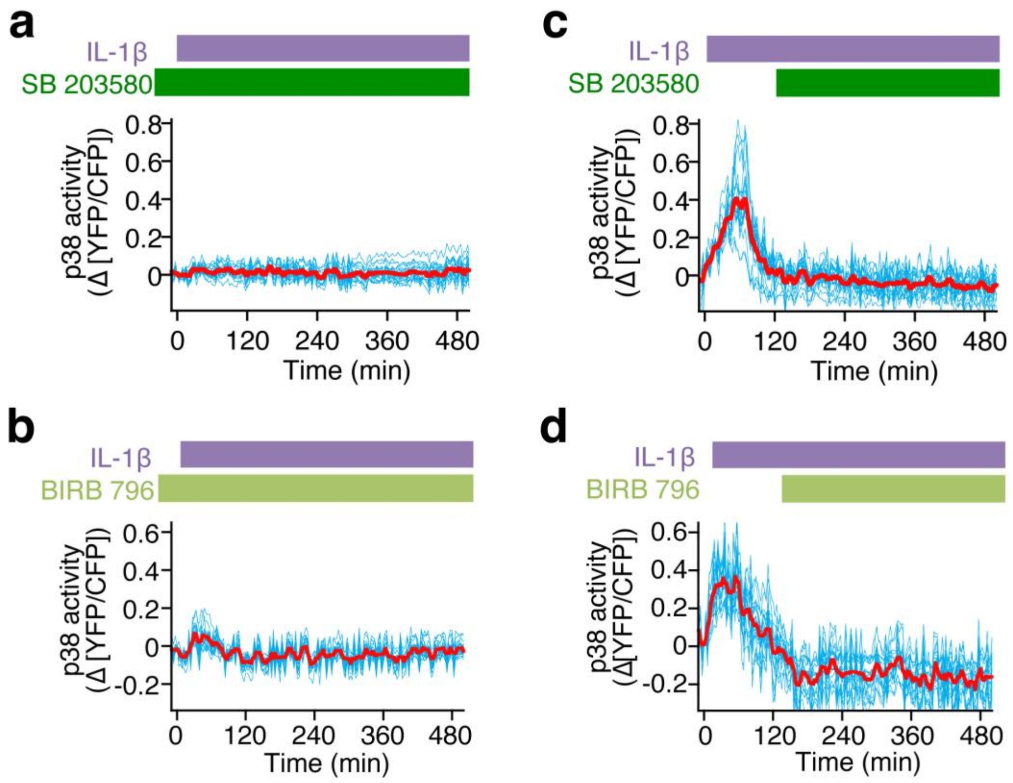

Supplementary Figure 4. Inhibition of the p38 reporter signal by p38 inhibitors.
p38 reporter cells were continuously stimulated with IL-1β (30 ng ml^{-1}) in the presence of 8 μM SB203580 (**a**, **c**) or 1 μM BIRB796 (**b**, **d**). Inhibitors were added 30 min before (**a**, **b**) or 2 h after (**c**, **d**) the onset of IL-1 β stimulation. Responses of individual cells are shown by cyan curves, and the average trace is in red (n=20 cells).

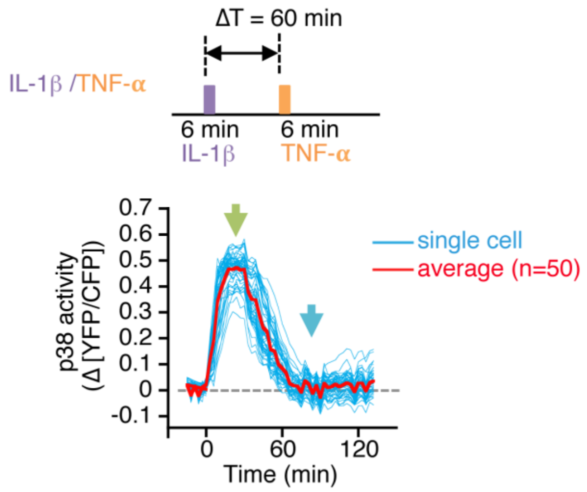

Supplementary Figure 5. p38 responses to paired-pulse stimulation using different cytokines. p38 reporter cells were first stimulated with IL-1β (6 min, 30 ng ml^{-1}), and, after 60 min, were stimulated with TNF-α (6 min, 50 ng ml^{-1}). Green arrow indicates the first peak of p38 activity, and cyan arrow indicates where the second peak would be expected. Responses of individual cells are shown by cyan curves, and average traces are in red (n=50).

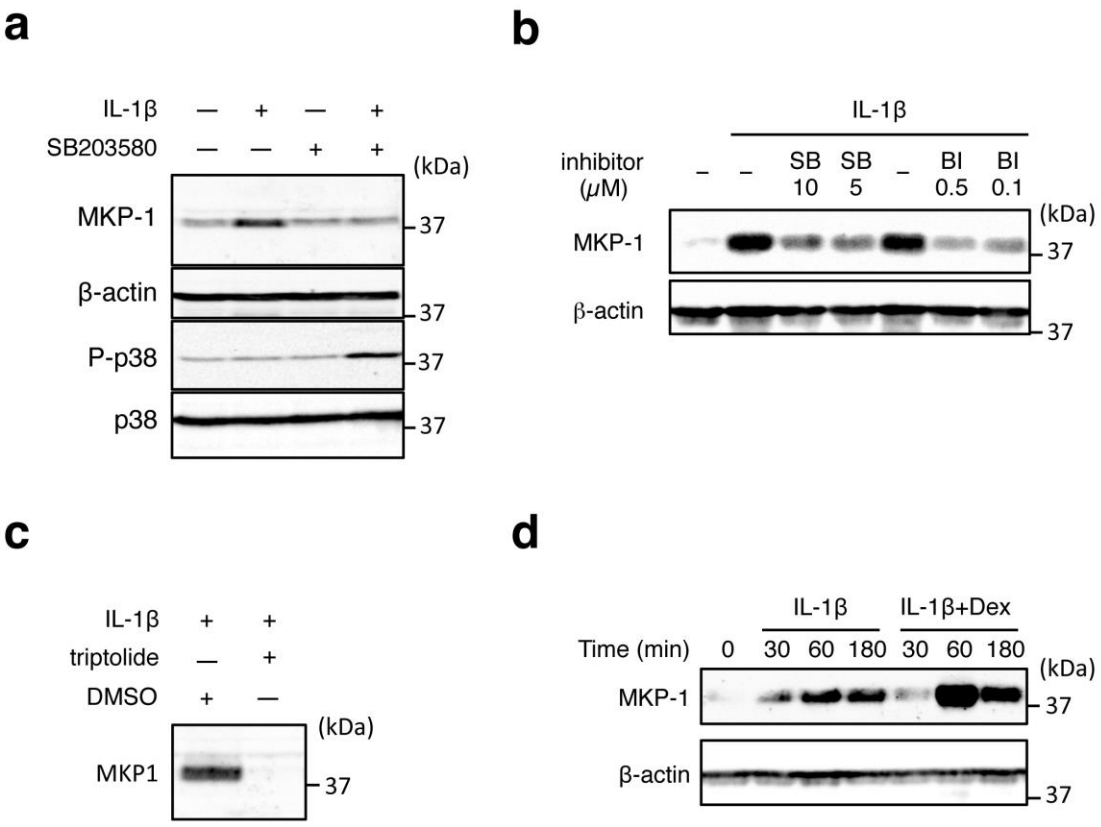

Supplementary Figure 6. Effects of pharmacological agents on the IL-1β-induced MKP-1 expression.

(a-b) Inhibition of the IL-1β-induced MKP-1 expression by the p38 activity inhibitors SB203580 and BIRB796. HeLa cells were stimulated with IL-1β (30 ng ml^{-1}, 70 min) in the presence or absence of 25 μM SB203580 (**a**) or indicated concentrations of SB203580 (SB) or BIRB796 (BI) (**b**). Expression of endogenous MKP-1 protein and the phosphorylation status of p38 were probed by immunoblot analyses. β-actin was a loading control. Note that SB203580 inhibits the kinase activity of p38, but does not inhibit p38 phosphorylation by MAP2K. Since SB203580 inhibits MKP-1 expression, the amount of p38 phosphorylation is actually elevated by this drug.

(c) Inhibition of the IL-1β-induced MKP-1 expression by triptolide. HeLa cells were stimulated with IL-1β (30 ng ml^{-1}, 60 min) in the presence or absence of triptolide (3 μM). Immunoblot analysis was performed using the anti-MKP-1 antibody.

(d) Enhancement of the IL-1β-induced MKP-1 expression by dexamethasone. HeLa cells were stimulated with IL-1β (30 ng ml^{-1}) for the indicated times in the presence or absence of 1 μM dexamethasone. Immunoblot analysis was performed using the anti-MKP-1 antibody.

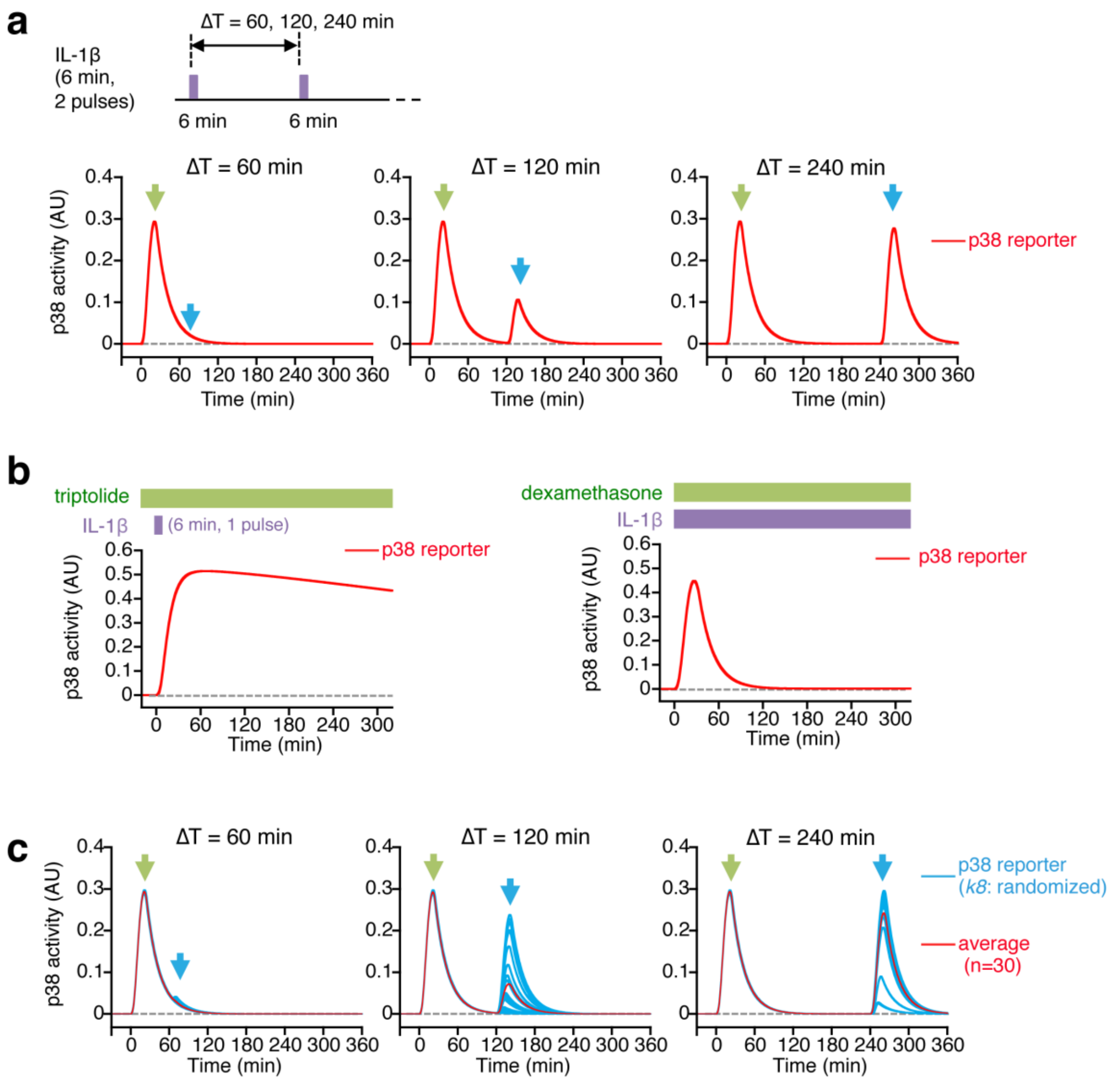

Supplementary Figure 7. Simulation of p38 activation.

(a) Simulation of p38 response to paired-pulse stimulation with different times between the pulses. Simulated p38 reporter responses are indicated (red lines) for easy comparison with imaging data. Green and cyan arrows indicate timings of the 1st and 2nd peaks, respectively.

(b) Simulation of the p38 response to IL-1β in the presence of triptolide or dexamethasone, using the activation protocols in Fig. 2e and 2g, respectively. In this model, the effect of triptolide was simulated by a decreased rate of MKP-1 expression, whereas the effect of dexamethasone was simulated by a constant elevated MKP-1 expression rate. Red lines indicate simulated p38 responses.

(c) Simulation of single-cell level variability in p38 activation. p38 response to paired-pulse stimulation was simulated. The value for *k8* was randomly varied within the range from 0.004 to 0.036. Individual traces (cyan) correspond to predicted single cell responses with different *k8* values. The red lines are averages of 30 traces. The green and cyan arrows indicate the first and subsequent p38 activity response, respectively.

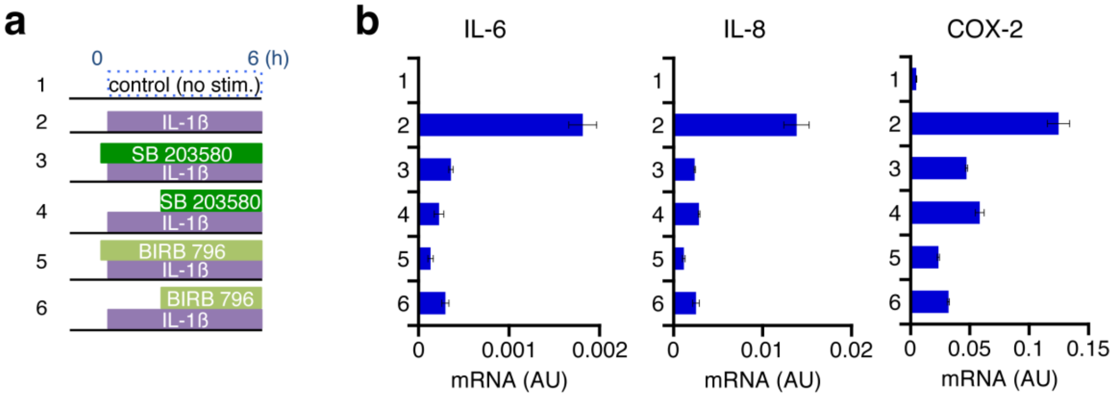

Supplementary Figure 8. Effects of p38 inhibitors on the IL-1β-induced pro-inflammatory gene expression.

(**a**) Diagrams of stimulation/inhibition protocols. HeLa cells were left unstimulated (protocol No. 1), or were stimulated with IL-1β (30 ng ml^{-1}) continuously for 6 h. In protocols No. 3 and No. 5, p38 inhibitors (8 μM SB203580 or 1 μM BIRB796) were added 30 min before the onset of IL-1β stimulation, whereas in protocols No. 4 and 6, inhibitors were added 2 h after IL-1β.

(**b**) The mRNA expression of pro-inflammatory genes was determined at the end of the 6 h stimulation protocols using real-time qPCR. Error bars represent SEM (n=3).

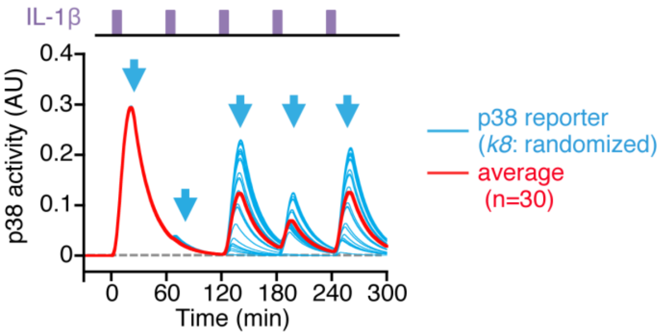

Supplementary Figure 9. Simulation of p38 activation by multiple pulse stimulation. Each stimulatory pulse lasted for 6 min, and the interval between the onsets of pulses was 60 min. The value for *k8* was randomly varied within the range from 0.004 to 0.036. Individual traces (cyan) correspond to predicted single cell responses with different *k8* values. The red lines are averages of 30 traces. The green and cyan arrows indicate the first and subsequent p38 activity response, respectively.

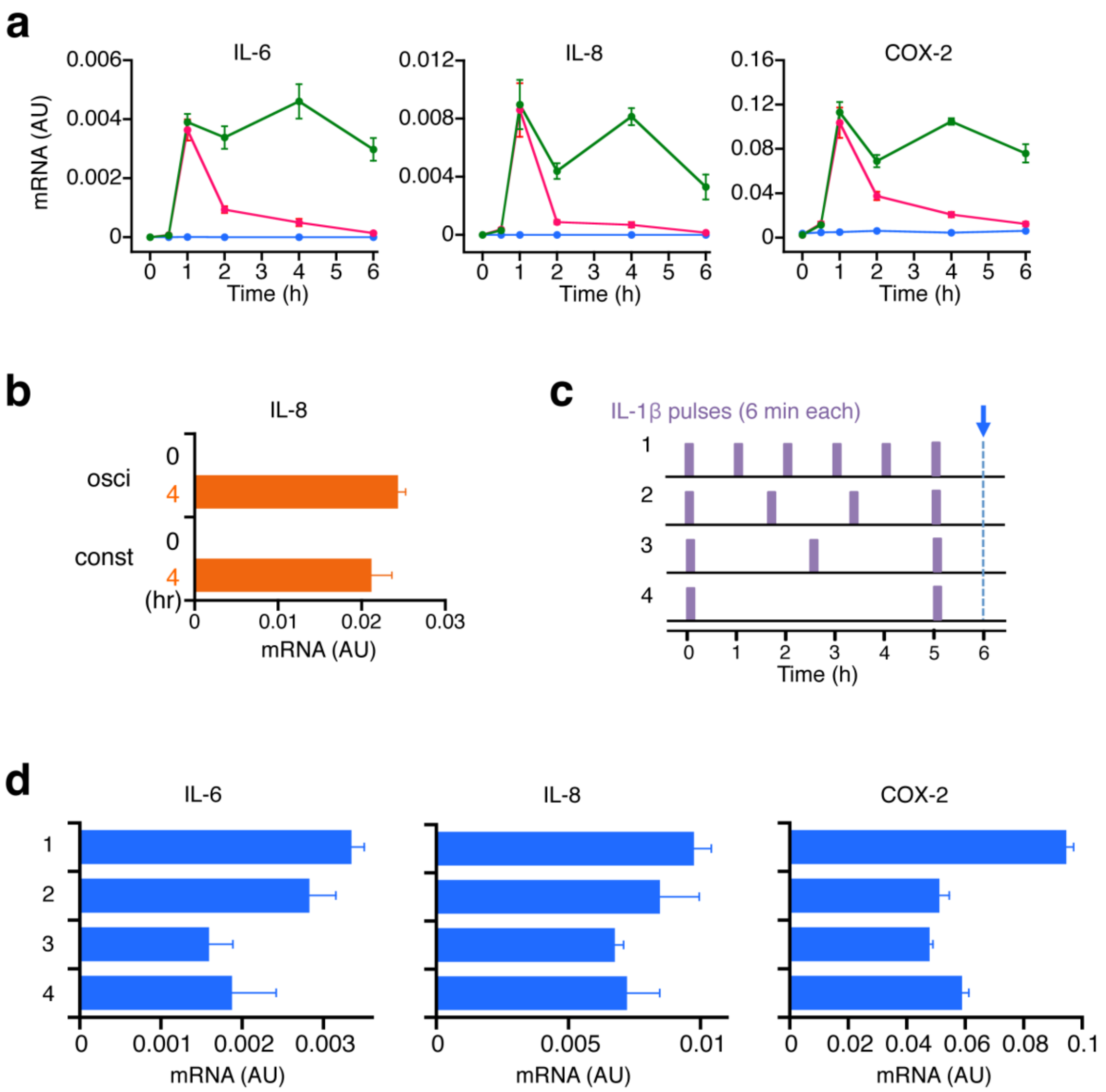

Supplementary Figure 10. Efficient expression of pro-inflammatory cytokine mRNA induced by repetitive IL-1β exposure.

(**a**) Time courses of pro-inflammatory cytokine mRNA expression. HeLa cells were stimulated by IL-1β using the No. 1 (blue), 5 (red) or 6 (green) protocol shown in Fig. 4a. Amount of mRNA was quantified by qPCR. Error bars represent SEM (n=3).

(**b**) Expression of IL-8 mRNA in HeLa cells stimulated by oscillatory IL-1β stimulation (No. 2 protocol in Fig. 4a) or constant IL-1β stimulation (No. 6 protocol). Amount of mRNA was quantified by qPCR before (0) and after 4 h (4) IL-1β stimulaiton. Error bars represent SEM (n=3).

(**c**) Diagrams of the stimulation protocols used in **d**. HeLa cells were stimulated with IL-1β (30 ng ml^{-1}) with repetitive 6 min pulses as indicated. Amount of mRNA was quantified at 6 h (blue arrow) by qPCR.

(**d**) Expression of pro-inflammatory cytokine mRNA in HeLa cells stimulated by IL-1β using the activation protocols shown in **c**. Amount of mRNA was determined at the end of the 6 h stimulation protocols using real-time qPCR. Error bars represent SEM (n=3).

Fig. 1a

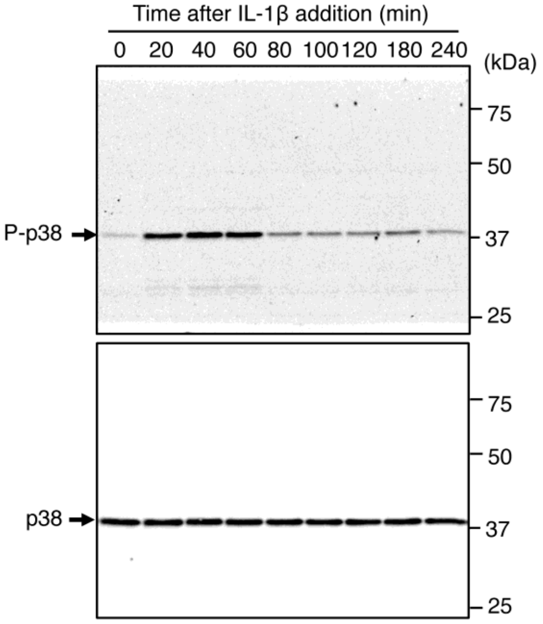

Fig. 2d

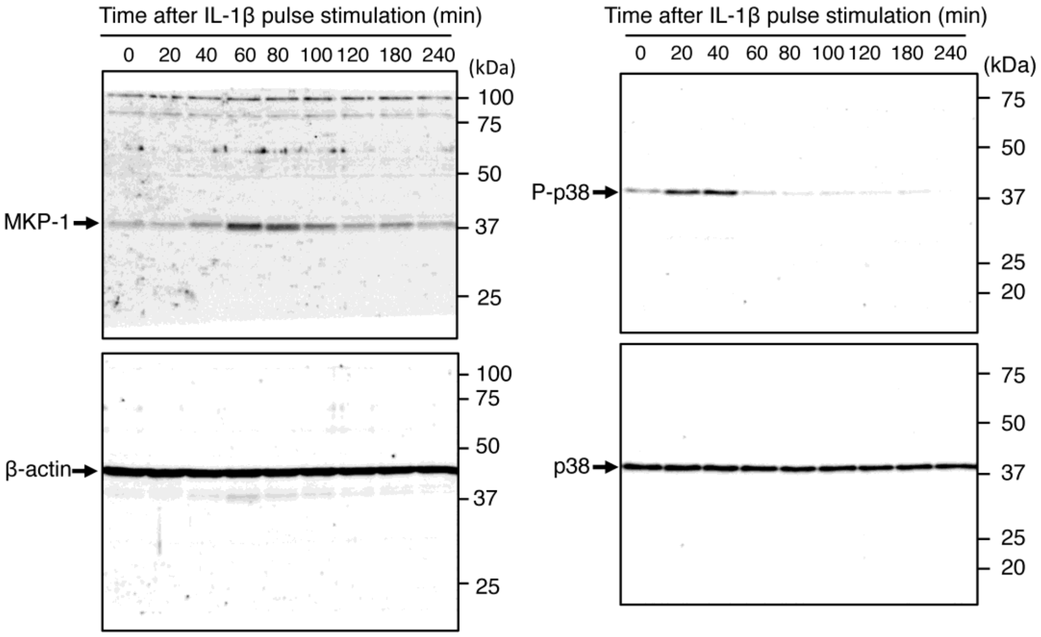

Supplementary Figure 11.
Uncropped scans of the immunoblots in Figures 1a and 2d.
